# Region Segmentation of Whole-Slide Images for Analyzing Histological Differentiation of Prostate Adenocarcinoma Using Ensemble EfficientNetB2 U-Net with Transfer Learning Mechanism

**DOI:** 10.3390/cancers15030762

**Published:** 2023-01-26

**Authors:** Kobiljon Ikromjanov, Subrata Bhattacharjee, Rashadul Islam Sumon, Yeong-Byn Hwang, Hafizur Rahman, Myung-Jae Lee, Hee-Cheol Kim, Eunhyang Park, Nam-Hoon Cho, Heung-Kook Choi

**Affiliations:** 1 Department of Digital Anti-Aging Healthcare, u-AHRC, Inje University, Gimhae 50834, Republic of Korea; 2 Department of Computer Engineering, u-AHRC, Inje University, Gimhae 50834, Republic of Korea; 3 JLK Artificial Intelligence R&D Center, Seoul 06141, Republic of Korea; 4 Department of Pathology, Yonsei University College of Medicine, Seoul 03722, Republic of Korea

**Keywords:** deep learning, prostate adenocarcinoma, segmentation, transfer learning, histological, U-Net

## Abstract

**Simple Summary:**

Differentiating growth patterns of the tumor glands in prostate biopsy tissue images is a challenging task for pathologists. Therefore, advanced technology, especially deep learning techniques, is needed to improve cancer diagnosis and reduce the workload of the pathologist. In this research work, we aimed to analyze whole-slide images of prostate biopsies and differentiate between stroma, benign, and cancer tissue components through deep learning techniques. Instead of image classification, we developed different deep CNN models for tissue-level prostate cancer adenocarcinoma histological segmentation. With these techniques, different patterns in a whole-slide image can be analyzed for cancer diagnosis.

**Abstract:**

Recent advances in computer-aided detection via deep learning (DL) now allow for prostate cancer to be detected automatically and recognized with extremely high accuracy, much like other medical diagnoses and prognoses. However, researchers are still limited by the Gleason scoring system. The histopathological analysis involved in assigning the appropriate score is a rigorous, time-consuming manual process that is constrained by the quality of the material and the pathologist’s level of expertise. In this research, we implemented a DL model using transfer learning on a set of histopathological images to segment cancerous and noncancerous areas in whole-slide images (WSIs). In this approach, the proposed Ensemble U-net model was applied for the segmentation of stroma, cancerous, and benign areas. The WSI dataset of prostate cancer was collected from the Kaggle repository, which is publicly available online. A total of 1000 WSIs were used for region segmentation. From this, 8100 patch images were used for training, and 900 for testing. The proposed model demonstrated an average dice coefficient (DC), intersection over union (IoU), and Hausdorff distance of 0.891, 0.811, and 15.9, respectively, on the test set, with corresponding masks of patch images. The manipulation of the proposed segmentation model improves the ability of the pathologist to predict disease outcomes, thus enhancing treatment efficacy by isolating the cancerous regions in WSIs.

## 1. Introduction

The fifth leading cause of cancer-related deaths worldwide and the most prevalent malignancy in males is prostate cancer [1,2]. Prostate cancer strikes more than 70% of men over the age of 70; 10% of these cases result in death [3,4,5]. To provide effective therapy, a deeper understanding of the kind and stage of prostate cancer is required. Therefore, precise predictive factor classification and segmentation for this malignancy are urgently needed. The Gleason score analysis of prostate biopsies is currently considered the industry-standard method for evaluating the cancer stage and progression.

The Gleason score measures the architecture of neoplastic glands using a five-grade system. It is a rather simple approach for determining the prognosis of prostate cancer and is widely used by pathology units worldwide. However, its repeatability is a problem. The subjectivity of the evaluation produces variations in assessment results, as cited in the literature [6,7,8]. In patients with a low Gleason score, follow-up has become a viable alternative to prostatectomy in recent years; as a result, the repeatability of this parameter is increasingly important, not only for determining the prognosis but also for choosing the best treatment strategy. In addition, the architectural pattern of neoplastic glands is the only factor taken into account when calculating the Gleason score; however, certain patterns have overlapping characteristics and may be evaluated incorrectly, leading to erroneous results [9,10,11]. Pathology units also spend a lot of time coming up with a Gleason score. For a single patient, pathologists must examine 12 biopsies and submit a report detailing the characteristics of cancer (e.g., including, at a minimum, the Gleason score, Gleason grade, biopsy length, tumor length, percentage of tumor-affected tissue, lesion continuity/discontinuity, and the ratio of affected to total cores) [12,13].

Digital prostate cancer and noncancer region segmentation may provide essential support for the Gleason score evaluation in this difficult environment. In the realm of pathology, the use of digital analysis and picture segmentation may help to more precisely identify particular structures, including prostatic glands, for enhanced pattern recognition [14,15]. Automatic image analysis assistance would serve to improve recognition and its consistency in both healthy and malignant areas and the interpretation of their arrangement. With this approach, the subjectivity issue would be resolved, improving the detection accuracy. To increase the precision and effectiveness of histopathological image analysis, several strategies have recently been put forth for the development of automated glandular segmentation algorithms [16,17]. Due to variations in the shape, size, and internal architecture of the prostate glands, particularly in disease situations, segmenting the prostate can be a difficult process. Using deep learning (DL) techniques, the detection of cancerous and noncancerous regions can be formulated as a direct segmentation problem.

In this study, we introduce an ensemble-based segmentation model—a modified version of EfficientNetB2 U-Net—to differentiate prostate cancer from benign tissue components. Moreover, we compared the performance of our method with existing pretrained models. The main aim of this work is to perform the region segmentation of different tissue components and build an auto annotation system (AAS) to assist pathologists in making consistent diagnoses. The patch extraction from WSIs and the procedures to train and test artificial intelligence (AI) algorithms are outlined in the Methods section.

## 2. Related Works

Numerous techniques for segmenting glandular structures have recently been put forth. Farjam et al. [18] combined Gaussian filters to recover the textural characteristics of prostate tissue; then, the borders of the glands were extracted using k-means clustering. When dealing with images that have large stain intensity fluctuations, texture-based techniques typically perform poorly and do not take into account the spatial information between epithelial nuclei and lumen. Different algorithms have been developed to take advantage of the link between nuclei/stroma and gland morphology to solve this issue. For example, a level set was used by Naik et al. [19] for prostate gland segmentation; a Bayesian classifier that recognized all of the lumen areas was able to identify the starting contour of the curve. Then, when the level set was close to the epithelial nuclei, an energy functional was applied to retrieve the minimal energy. The level set creates good segmentation results if applied correctly. However, its primary restriction is the initialization process. Specifically, the starting level may be incomplete or erroneous; thus, this family of algorithms may provide improper segmentation (e.g., in the case of glands with no visible lumen).

To identify the four primary components of histological tissue, Peng et al. [20] used color decomposition and principal component analysis; this was followed by a postprocessing technique to locate the gland borders. In healthy tissue, this technique can locate glands with fair accuracy; however, when tumoral patterns are present, the segmentation accuracy suffers noticeably. Nguyen et al. [21] performed the pixel categorization of nuclei, cytoplasm, lumen, and stroma based on information from the LAB color space. Then, the glandular areas were extracted using an approach that combined lumen, cytoplasm, and nuclei pixels. Stroma, gland, and lumen pixels were identified using a logistic regression classifier developed by Singh et al. [22]. The final segmentation of the glands involved a heuristic procedure. Notably, these techniques were capable of successfully segmenting glands with discontinuous or nonexistent lumen; however, they failed to do so for structures with a lumen (as in the case of pathological conditions).

DL techniques have recently attained cutting-edge performance in numerous medical imaging disciplines, including digital pathology image processing [23,24,25]. A convolutional neural network (CNN) for prostate gland segmentation in histopathology images was suggested by Ren et al. [26]. In this approach, an encoder network and a decoder network were used to execute semantic segmentation. In their setup, the encoding and decoding networks each had 10 convolutional layers, and the CNN’s input layer had dimensions of 480 × 360 × 3. Slider windows were used to segment larger images. Compared to earlier works, high performance was demonstrated with this approach. Using three CNN channels, Xu et al. [27] concentrated on gland segmentation. One channel was used to separate the background from foreground pixels. A second channel was used to identify gland borders, while the third channel was used to identify specific glands. To obtain the final segmentation result, the CNN fused the outputs of the three channels. Here, it is important to emphasize that although benign and nicely shaped glands are useful for gland detection, deep networks are capable of recognizing glands in situations of malignancy. Nevertheless, Soerensen et al. [28] proposed a deep neural network—ProgNet—to segment the prostate gland on magnetic resonance imaging (MRI) for targeted biopsies in routine urological clinical practice. They used T2 MRI scans to carry out this research and compared the performance with other DL networks. To measure the segmentation results, they used the Dice similarity coefficient (DSC). 

In this study, we used microscopic prostate biopsy images, WSIs, to perform tissue-level segmentation and differentiate cancer tissue components using DL techniques. However, we not only performed segmentation but also developed AAS for WSI analysis. 

## 3. Materials and Methods

### 3.1. Dataset

**Figure 1:**
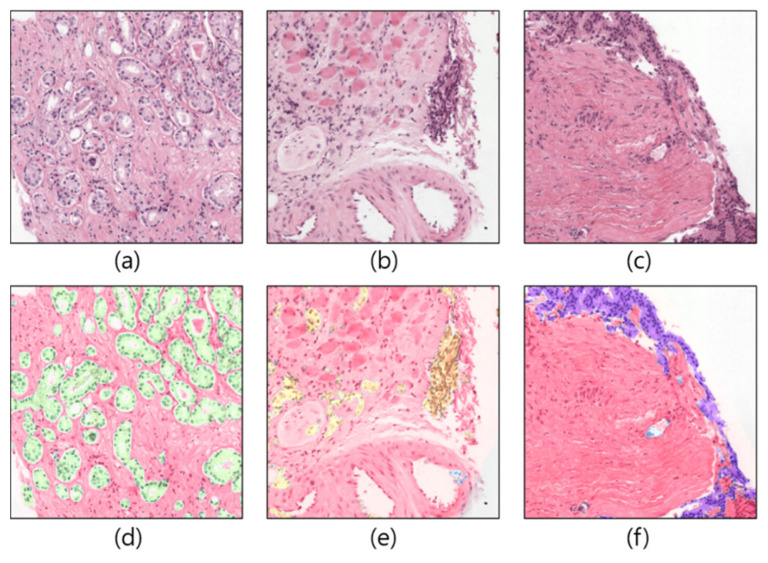
Example of patch images cropped from WSIs. (**a**–**c**) Original samples. (**d**–**f**) Corresponding ground-truth samples.

**Figure 2:**
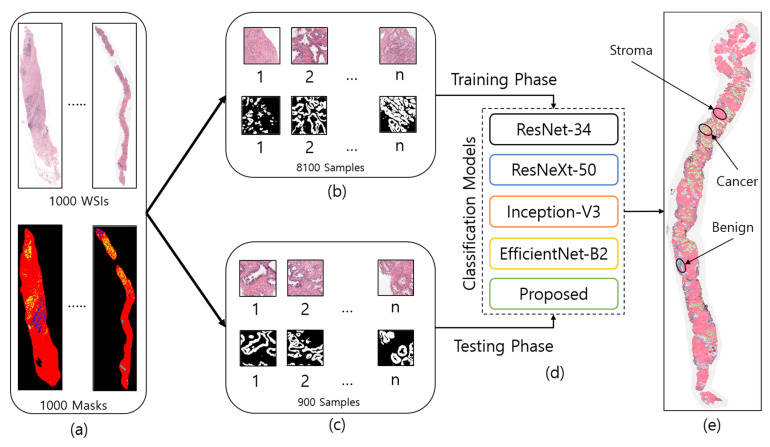
The entire process of region segmentation of WSIs for diagnosis of Prostate Adenocarcinoma. (**a**) The entire dataset of whole-slide images and ground-truth samples. (**b**) Patch images for training. (**c**) Patch images for testing. (**d**) Classification models for training and testing. (**e**) WSI prediction and auto annotation of stroma, benign, and cancer regions.

The dataset used in the current study was downloaded from the Kaggle repository, accessible at https://www.kaggle.com/c/prostate-cancer-grade-assessment (accessed on 28 July 2022). Figure 1 shows a few examples of patch/tile images and their corresponding masks, which are cropped from the original and annotated WSIs, respectively. A total of 1000 WSI samples were used in this research to develop AAS using prostate biopsy tissue images that were analyzed at the Radboud University Medical Center in Nijmegen, Netherlands. The dataset was uploaded to the Kaggle repository by Bulten et al. [29] for the Prostate Cancer Grade Assessment (PANDA) competition. Based on each patient’s pathology report, a single hematoxylin and eosin (H&E)-stained glass slide containing the tumor’s most aggressive portion was chosen for scanning [30]. A 3DHistech Panoramic Flash II 250 scanner (3DHistech, Budapest, Hungary) was used to scan each of the chosen glass slides at 20× magnification. From 1000 WSIs, we cropped 8100 patch images for training and 900 for the testing phase, as shown in Figure 2.

### 3.2. Image Preprocessing

**Figure 3:**
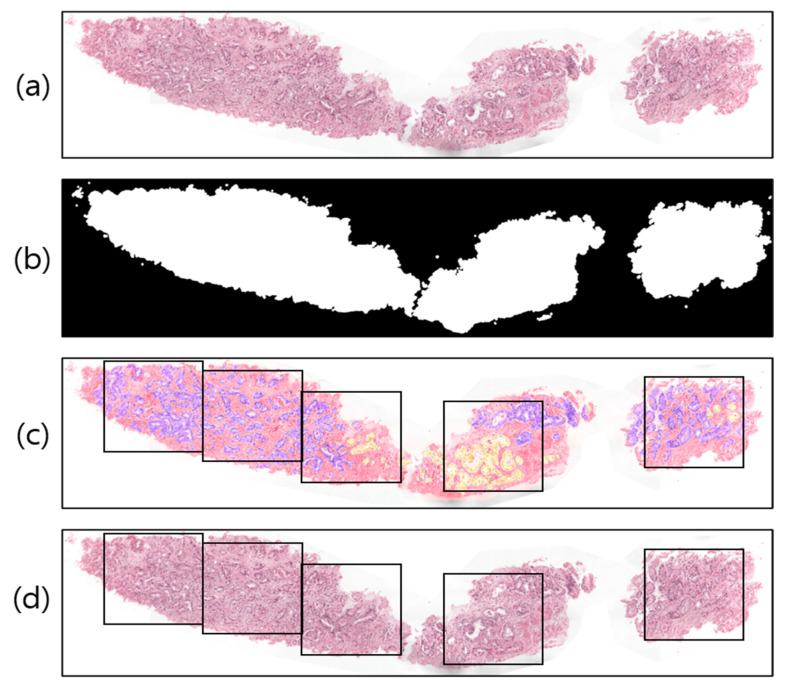
Examples of image preprocessing. (**a**) Original WSI. (**b**) Threshold result of (**a**). (**c**) Pathologist-annotated/ground-truth image with several nonoverlapping blocks for patching. The violet and yellow colors represent tissue regions with Gleason scores of 4 and 5, respectively. (**d**) The result of patching from the original WSI.

A WSI is a large-volume digital representation of a microscopic slide. A microscope scans a slide and assembles tiny pictures into a larger image, sometimes known as a gigapixel image. The gigapixel image is too large to fit on a GPU all at once. Therefore, we used patch images for region segmentation. To prepare the WSI sample (Figure 3a) for tiling/patching, the RGB image was split into two levels by one threshold, 0 < T < 255, a procedure known as global thresholding [31]. To achieve the desired outcome (a binary image with one bit per pixel), the threshold value was manually adjusted to T = 210, rather than being calculated automatically. Figure 3b illustrates this concept by designating the intensities of the pixels above the threshold T as foreground pixels (i.e., labeled 1) and the intensities of the pixels below the threshold T as background pixels (i.e., labeled 0).

Image tiling is a crucial phase in the WSI analysis process. Figure 3c shows an example of a pathologist-annotated slide, in which the violet and yellow colors signify Gleason scores of 4 and 5, respectively. However, depending on the x and y coordinates of the foreground pixels (Figure 3b) and sliding window (ix,iy) in Figure 3d, we cropped the images of size 256 × 256 pixels into several nonoverlapping blocks with grid spacing ix = jy = 256 along both rows and columns from the original WSI. Similarly, image patching was also carried out from the annotated slides (Figure 3c) to train the segmentation models. The patches extracted from the WSI tissue regions correspond to five classes, namely, stroma, benign, score 3, score 4, and score 5. To train the network, we sorted the patches and considered score 3, score 4, and score 5 as the cancer class; the two other classes remained the same: stroma and benign. Thus, ultimately, there were three classes in total: stroma, benign, and cancer.

### 3.3. Tissue Region Segmentation

Region segmentation is of paramount importance for classifying stroma, benign, and cancer tissue components in WSIs. Training a model from scratch is a difficult task, as it is computationally expensive and requires that the parameters be changed several times based on the learning performance. Therefore, the most common transfer learning method was employed in this study. Specifically, we leveraged popular DL models that were pretrained on the ImageNet dataset [32] as backbones of segmentation architectures to perform feature extraction in the encoding path of the network.

#### 3.3.1. Transfer Learning

**Figure 4:**
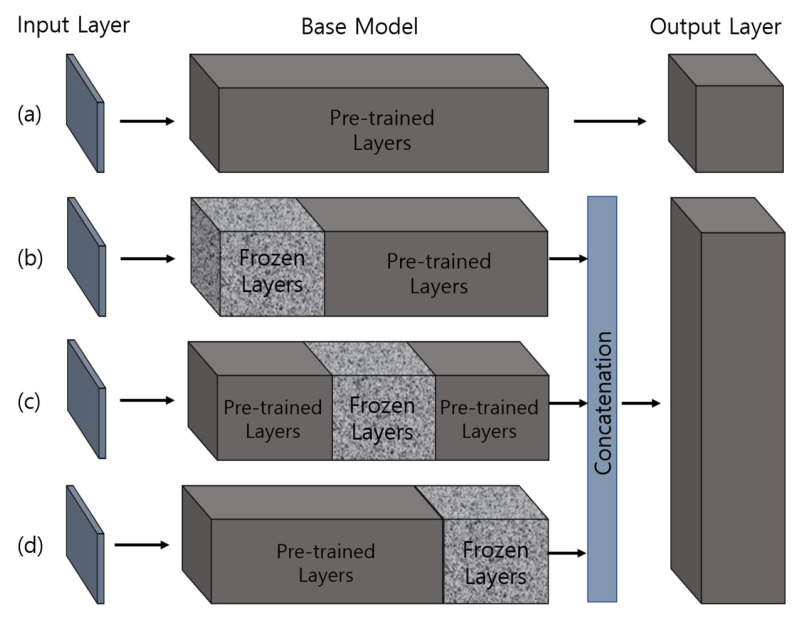
Approaches for training pretrained models based on transfer learning. The weights of pretrained layers in the base models are modified during the learning process, whereas the frozen layers retain their weights (i.e., not modified). (**a**) A basic transfer learning approach, where the pretrained model is loaded and trained using the extracted features. (**b**–**d**) The proposed transfer learning approach, where three freezing techniques are applied and features from each model are concatenated to perform training.

Transfer learning [33] is powerful, as it allows the neural network to handle small inputs for creating a new domain while transferring a sizable preexisting dataset to the task, thus minimizing the time and computational costs. Medical image datasets have limited amounts of labeled data. For managing the bare minimum of medical data, transfer learning offers the ideal solution, as it dramatically accelerates the training process and reduces the computational cost of the network. In addition, freezing [34] and fine-tuning [35] techniques can be applied to further modify the DL model to achieve even better accuracy. Figure 4 shows the implementation strategies of transfer learning for training segmentation models.

#### 3.3.2. Network Architecture

**Figure 5:**
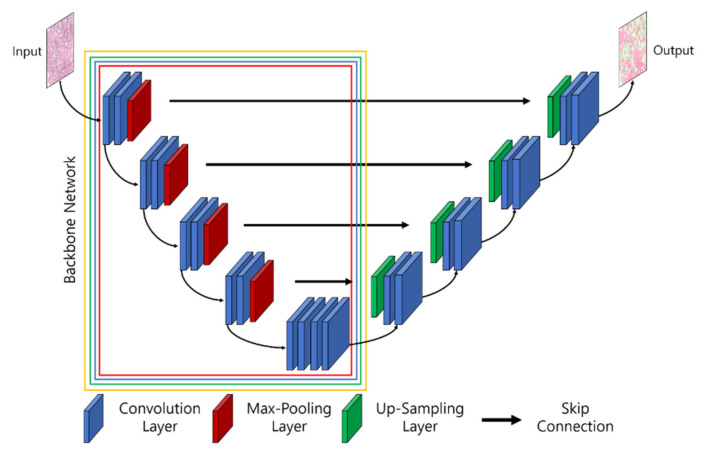
The architecture of UNet and the pretrained convolution neural network (i.e., backbones for feature extraction). The parts of the backbone network marked with red, blue, green, and yellow signify Resnet34-UNet, ResNeXt50-UNet, InceptionV3-UNet, and EfficientNetB2-UNet.

To segment biomedical images, different network architectures have been developed by researchers, such as UNet, UNet+, SegNet, SegNet-UNet, SegNet-UNet+, ResNet-UNet, attention UNet, and so forth [36,37,38]. In this study, we introduce an ensemble segmentation model based on transfer learning strategies. Five pretrained segmentation models, namely, UNet, ResNet34-UNet, ResNeXt50-UNet, InceptionV3-UNet, and EfficientNetB2-UNet, were adapted for experimental purposes and compared to the proposed ensemble segmentation model. Here, ResNet-34, ResNeXt-50, Inception-V3, and EfficientNet-B2 are backbones of UNet and refer to the base models. Figure 5 shows the architecture of the combined UNet and pretrained segmentation models, in which the input consisted of extracted patch images $x\in R^{256\times 256\times 3}$ and the output was the segmented mask $y\in R{\left(0,\;1,\;2\right)}^{256\times 256\times 3}$ of three classes: stroma, benign, and cancer. A description of the segmentation models used is given in the following.

**Figure 6:**
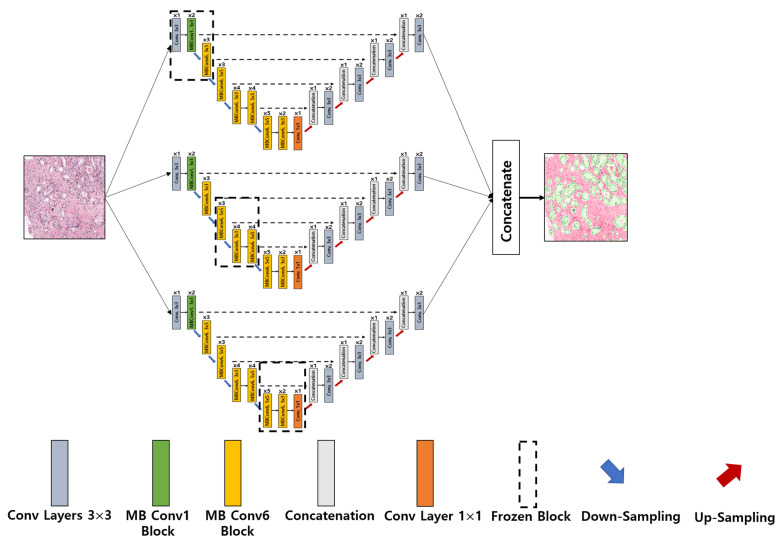
Implementation of the proposed ensemble-based segmentation model. Conv: convolution; MB Conv: mobile inverted bottleneck convolution.

1. UNet Architecture: UNet [39] was initially proposed and implemented in 2015. The network consists of encoder and decoder convolutional blocks, with skip connections and a bottleneck layer to propagate encoded features to decoder blocks. The encoder includes a number of blocks, each of which accepts input using two 3 × 3 convolution layers, followed by a rectified linear unit (ReLU) and 2 × 2 max-pooling layers, which down-sample the image by 2 for the next layer. The obtained feature maps are propagated to the decoder block through the bottleneck layer to convert a vector into a segmented image and up-sample the image with 2 × 2 up-convolutional layers. Between the layers of contraction and expansion, the bottleneck layer causes interference. The context is captured via a compact feature map using an encoder-like contraction route.
2. ResNet-34 UNet Architecture: ResNet-34 [40] is a CNN architecture made up of a number of residual blocks. It varies from other CNNs by having shortcut connections [41]. This technique is used in residual building blocks to skip the convolutional layers. The final calculation is performed by adding the input features with a residual block output via a skip link. Therefore, the problem of a vanishing gradient is alleviated by increasing the depth of the neural network. When introduced, ResNet easily won that year’s ImageNet competition. Figure 5 shows ResNet34 as a backbone model of UNet [42,43]. The residual block is constructed using several convolutional layers (Conv), batch normalization (BN), a ReLU activation function, and a shortcut. Similarly, the entire ResNet34 is constructed using 33 convolutional layers, a max-pooling layer of size 3 × 3, and an average pooling layer followed by a fully connected layer.
3. ResNeXt50 UNet Architecture: RexNeXt, a variant of ResNet, was developed in 2017 by Xie et al. [44]. The primary distinction between ResNeXt and ResNet is that instead of having continuous blocks (one after the other), ResNeXt considers and integrates ‘cardinality’ or the size of transformations, drawing inspiration from Inception/GoogleLenet. Notably, ResNeXt outperformed ResNet in the Imagenet Challenge, despite having fewer parameters. On the same dataset, the prior U-Net design that had been enhanced with the ResNeXt50 backbone was put into practice. The concept behind ResNeXt is that it combines several transformations with similar topologies using repeating building elements. Experimenting with cardinality (the size of the collection of transformations) gives depth and width further advantages. As a result, the network’s accuracy can be increased more efficiently by increasing cardinality. This makes it possible to explore the dataset while also upgrading the underlying U-Net design using ResNeXt50 blocks.
4. InceptionV3 UNet Architecture: In 2014, Szegedy et al. launched GoogleNet, commonly known as Inception [45]. At the time, it was one of the biggest and most effective categorization networks. GoogLeNet/Inception is more computationally efficient than VGG in terms of parameters and costs, which include memory and other resources. It also lowered the network classification top-5 error rate to 6.67%. GoogLeNet/Inception comes in a number of iterations, including Inceptionv1, Inceptionv2, Inceptionv3, Inceptionv4, and Inception-ResNet. The version employed in this study, Inceptionv3, was applied to improve the network accuracy while lowering computational costs. Inceptionv3 is made up of 42 layers and around 24 million parameters. The network makes use of the multilevel feature extractor known as the inception block. The conception block is made up of filters of various sizes, including 1 × 1, 3 × 3, and 5 × 5. A convolutional layer with a filter size of 1 × 1 is utilized in the network’s center to minimize dimensionality, and global average pooling is used in place of completely linked layers.
5. EfficientB2 UNet Architecture: EfficientNet [46] is a novel CNN and scaling technique that uses a compound coefficient to consistently scale the depth, breadth, and image resolution. The EfficientNet scaling approach evenly scales the network breadth, depth, and resolution using a set of preset scaling coefficients, in contrast to standard practice, which scales these variables arbitrarily. The network width, depth, and resolution are all consistently scaled by EfficientNet logically using a compound coefficient. At first, EfficientNet-B0 was developed as a baseline network from Neural Architecture Search (NAS) using the AutoML MNAS framework [46]. Later, this baseline was extended and improved to obtain an efficient family (i.e., EfficientNet-B1-EfficientNet-B7). In general, EfficientNet is constructed using the mobile inverted bottleneck (MBConv) building block [47].
6. Ensemble EfficientNetB2 U-Net Architecture: The major sources of inspiration for this research were Ensemble Learning and UNet with EfficientNet-B2. To construct the proposed ensemble UNet model, we employed a multi-head pretrained CNN (i.e., EfficientNet-B2) to encode the feature maps from the input image and applied a fine-tuning technique, ‘freezing,’ to accelerate neural network training by progressively freezing hidden layers. Freezing a layer in the CNN is about controlling the process of updating the weights during backpropagation; specifically, if any layer is frozen, then its weight is not updated during model learning. In this study, we applied three freezing techniques to perform feature extraction in the encoder blocks, as shown in Figure 4b–d. The decoder blocks received the encoded feature maps to up-sample the image size, and the up-sampling output was concatenated with the output of the corresponding part of the encoder. The output of each fine-tune-based EfficientNetB2-UNet architecture was concatenated to create the final output of the proposed ensemble model, as shown in Figure 6. The encoder block (five down-sampling layers) contained a 3 × 3 convolutional layer, 22 MBConv structures, and a 1 × 1 convolutional layer (Table 1). The decoder block (four up-sampling layers) was constructed using up-sampled, concatenated, 3 × 3 convolutional layers.

## 4. Results and Discussion

In this section, the performance of the proposed method is demonstrated using the prostate biopsy dataset. This study primarily segmented tissue samples to segment cancerous and noncancerous regions. The dataset from the Radboud University Medical Center was analyzed, preprocessed, and separated for training and testing. The models were trained and tested on the samples of size 256 × 256 pixels at 10× magnification. Experiments were conducted using pretrained U-Net models for segmenting the tissue regions, and the results were evaluated using the DC, IoU [48], and Hausdorff distance [49] performance metrics to quantify the degree of overlap between ground truth and prediction regions. Furthermore, a comparative analysis was carried out between the proposed and existing methods.

### 4.1. Region Segmentation Results

The region segmentation performance was evaluated by comparing the proposed ensemble segmentation model with several existing architectures, such as ResNet- 34, ResNeXt-50, Inception-V3, and EfficientNet-B2. In image segmentation, the values of true positives (TP), false positives (FP), and false negatives (FN) are considered areas or the number of pixels. Model training was performed several times by configuring the different parameters at epoch = 50. The early stopping regularization parameter was used to prevent the overfitting and underfitting of the model and achieved the optimal results at epoch = 10, because the model performance stopped improving on a hold-out validation dataset. Table 2 shows the quantitative results of tissue region segmentation and the comparative analysis of the experimental methods. The DC, IoU, and Hausdorff distance metrics used for model evaluation can be expressed as:

(1) $$\frac{2\times TP}{\left(TP+FP\right)+\left(TP+FN\right)}$$

(2) $$\frac{\mathrm{TP}}{\left(\mathrm{TP}+\mathrm{FP}+\mathrm{FN}\right)}$$

(3) $$\delta_{H}\left(A,B\right)=max\left\{{\vec{\delta }}_{H}\left(A,B\right),\;{\vec{\delta }}_{H}\left(B,A\right)\right\}$$

The distance measure is symmetric. Here, A is the ground truth, B is the segmented mask, ${\vec{\delta }}_{H}\left(A,B\right)$ is the directed Hausdorff distance, typically the Euclidean distance, and it is not symmetric; ${\vec{\delta }}_{H}\left(B,A\right)$ is the reverse directed Hausdorff distance which is different from ${\vec{\delta }}_{H}\left(A,B\right)$. To compare the shape, we used undirected Hausdorff distance $\delta_{H}\left(A,B\right)$ that can be calculated as the maximum of the two directed distances.

From Table 2, the proposed ensemble segmentation model outperformed all others, giving an overall DC, IoU, and Hausdorff of 0.891, 0.811, and 15.9, respectively. Moreover, the proposed model surpassed ResNet-34, ResNeXt-50, Inception-V3, and EfficientNet-B2 by 8.9%, 1.9%, 4.8%, and 1.4%, respectively, in average DC scores; 12.2%, 3.1%, 6.8%, and 2.2%, respectively, in average IoU scores; and 1.6, 1.0, 0.5, and 1.2, respectively, in average Hausdorff.

**Figure 7:**
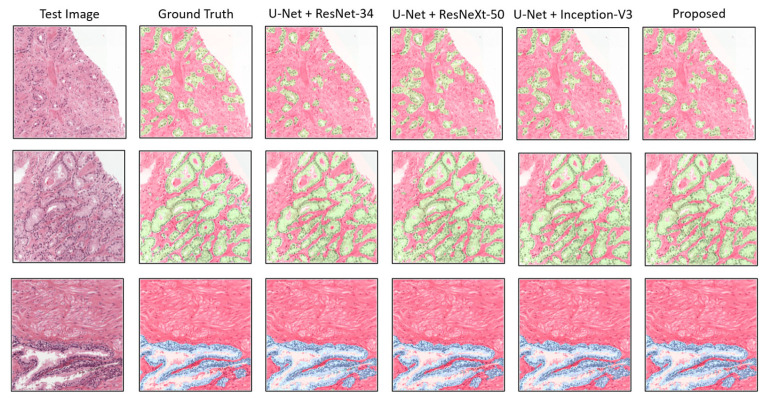
Comparison of segmentation examples from the proposed model with existing methods. Red, blue, and green colors signify stroma, benign, and cancer regions, respectively. The size of each tile in test set is the same as the train dataset, which is 256 × 256 pixels at 0.5μm/pixel.

Apart from the quantitative results, to show the qualitative performance of the experimental segmentation models, we plotted the patch-level prediction results, as shown in Figure 7. Here, several test images are presented with their ground truth and predicted masks for each class, such as stroma + cancer (first and second rows) regions and stroma + benign (third row) regions. For a clear representation, the segmented areas are marked with different colors in the figure, in which red is stroma, blue is benign, and green is cancer. No major differences in segmentation were evident in a comparison of the proposed method with the others. Nevertheless, differences were indicated in the quantitative results. The proposed model efficiently segmented tissue regions (i.e., stroma, benign, and cancer) without losing necessary information.

### 4.2. Slide-Level Prediction

**Figure 8:**
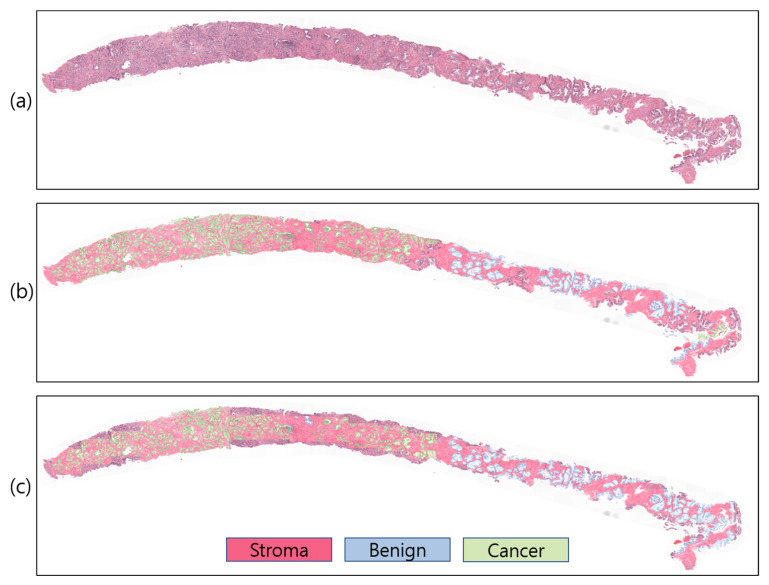
Slide-level prediction (stroma, benign, and cancer). (**a**) Original WSI. (**b**) Annotated WSI. (**c**) Predicted WSI. The slide-level visualization is carried out at 10× magnification.

To study and comprehend how a model segments and makes decisions for a particular job, the visualization technique is crucial. However, comparing projected outcomes is also crucial for determining model performance with respect to ground-truth data. For WSI analysis, several visualization methods exist. However, to visualize predicted probabilities, the predicted patch images are overlapped on the WSI using different colors to signify the stroma, benign, and cancer regions with white, red, blue, and green, respectively (Figure 8). In our study, the proposed ensemble segmentation model revealed representative information after iteratively learning from the patch-level diagnosis (Figure 8c). We also compared the predicted results with pathologist-annotated slides (Figure 8b) to validate the model performance. Our model performed well, similar to the ground-truth slides, from which we can say that the WSI segmentation of cancerous and noncancerous regions was successfully carried out using the proposed ensemble segmentation model.

DL has advanced significantly in several areas, including computer vision, producing impressive outcomes [50]. Due to the abundance of labeled datasets, including ImageNet, DL has also demonstrated effectiveness in medical image analysis. However, a CNN needs large amounts of training data before it can be successfully applied to any assignment. The lack of image datasets in medical disciplines frequently affects many researchers. This sparked our interest in the subject and ultimately led us to transfer learning, a different strategy from DL. By fine-tuning the fully connected layers to meet the needs of each job, transfer learning makes use of a model that has already been trained. Any learned model can be used as the foundation model for a new assignment. If the GPU is not powerful enough, training a CNN can take quite a while. Nevertheless, with transfer learning, one can train and categorize images in a matter of hours. In this work, we initially preprocessed WSIs to obtain patch images with 256 × 256 pixels for segmentation using several AI-based CNN models. 

We used patch-level analysis rather than slide-level to simplify prostate cancer region-based segmentation, which can save on computational costs and improve the efficiency of DL models in data analysis. To categorize the tissue samples that were taken from the WSI and carry out the automated identification/segmentation of cancerous and noncancerous areas, we employed several pretrained CNN models. To accomplish ensemble segmentation using the advantages of transfer learning, we presented a customized pretrained CNN model (Figure 6). With this approach, we were able to expedite neural network training by gradually freezing the hidden layers using several freezing techniques for each CNN model. We have studied numerous research articles related to prostate cancer classification using histopathological images. 

Most researchers differentiate cancerous from noncancerous tumors based on the features learned from the whole image. Ryu et al. [51] proposed the Gleason scoring system based on deep CNN for the diagnosis of prostate biopsy samples. They collected data samples from two different institutions that were digitized at 40× optical magnification. The segmentation network was trained on a set of 940,875 patch images of size 352 × 352 pixels extracted from 1133 WSIs. However, in our case, we used WSIs that were digitized at 20× optical magnification. The main difference between our and their work is that they used an internal dataset and developed an automatic Gleason scoring system using a huge number of tissue samples that were trained on eight-GPU machines, whereas we carried out region segmentation and built AAS to distinguish between stroma, benign, and cancer tissue components, which can reduce the complexity of WSI analysis for pathologists. We used different segmentation networks to perform a comparative analysis, and all of the networks were trained on a set of 8100 patch images of size 256 × 256 pixels on a one-GPU machine.

Histopathology images are usually large (about 40,000 × 40,000 pixels). Because of limited memory, the models were trained on a set of patch images to reduce memory consumption and increase computational time. However, hyperparameter selection before the training process is also important for handling GPU memory; higher batch size values can lead to higher memory consumption. Therefore, keeping all of the requirements in mind, we set a batch size = 8 to train the model efficiently. Additionally, the models were not trained for all epochs; we used a function from the TensorFlow library—early stopping—to monitor the validation loss and stop the training process if the loss failed to decrease for a sequence of five epochs.

WSI annotation is commonly required in supervised classification for cancer grading, which can be time-consuming and impractical in everyday pathology. The dataset we used in this study, however, consisted of both WSIs and annotated images that were examined by pathologists at the Radboud University Medical Center and whose reports (from between 1 January 2012, and 31 December 2017) were retrieved for patients who underwent a prostate biopsy, as they were suspected of having prostate cancer. As a result, the pathologists extensively annotated the WSIs used in this study. From this, we created an automated AI-based computer-aided segmentation system to successfully perform patch-level analysis region segmentation in WSIs.

Some limitations have been observed in this study. First, the models were not trained with a sufficient amount of data due to GPU and memory issues. Second, we do not have an internal dataset with accurate annotations to train and test the models. Third, we used an existing model and modified it for region segmentation based on the transfer learning technique instead of developing a state-of-the-art deep learning model. Lastly, the patches were extracted from WSIs at low magnification levels, which can cause information loss.

## 5. Conclusions

Using whole-slide histopathology images, we created an automated computer-aided segmentation system. In the coming decades, pathology practice will surely change as a result of gigapixel WSI analysis and grading that employs AI techniques. We applied pretrained and newly developed CNN models for multiclass segmentation to perform patch-level analysis and WSI segmentation. However, to analyze the WSI sample and divide it into three segmentation groups (stroma, benign, and cancer), several methodologies were applied. The WSIs were divided into a sequence of nonoverlapping image blocks for patch-level analysis using two preprocessing approaches (global thresholding and image tiling). An ensemble-based pretrained model was developed to predict and grade the WSIs after being trained at the patch level. Our proposed model performed better than other pretrained CNN models when tested on 900 patch images that were not used in the training phase. Looking forward, we intend to expand this research work to enhance the proposed CNN model or develop a new robust method for real-time WSI prediction and annotation.

## Figures and Tables

**Table 1:** Encoder specification for the proposed segmentation model.

Steps	Operator	Resolution	Layers
Down-sampling (1)	Conv 3 × 3, Stride = 2	128 × 128	1
	MBConv1, 3 × 3	128 × 128	2
Down-sampling (2)	MBConv6, 3 × 3	64 × 64	3
Down-sampling (3)	MBConv6, 5 × 5	32 × 32	3
Down-sampling (4)	MBConv6, 3 × 3	16 × 16	4
	MBConv6, 5 × 5	16 × 16	4
Down-sampling (5)	MBConv6, 5 × 5	8 × 8	5
	MBConv6, 3 × 3	8 × 8	2
	Conv 1 × 1	8 × 8	1

**Table 2:** Comparative analysis of the performance of the proposed model with some existing methods based on Dice score, IoU, and Hausdorff on a test dataset.

		ResNet34	ResNeXt50	InceptionV3	EfficientNetB2	Proposed
Stroma	Dice coefficient	0.935	0.949	0.947	0.949	0.956
	IoU	0.878	0.903	0.900	0.903	0.916
	Hausdorff (mm)	17.7	16.9	16.6	17.2	15.8
Benign	Dice coefficient	0.624	0.775	0.705	0.778	0.802
	IoU	0.453	0.633	0.544	0.638	0.670
	Hausdorff (mm)	17.4	16.9	16.4	17.3	16.2
Cancer	Dice coefficient	0.848	0.892	0.879	0.905	0.914
	IoU	0.736	0.805	0.785	0.827	0.843
	Hausdorff (mm)	17.5	16.9	16.3	16.9	15.9
Average	Dice coefficient	0.802	0.872	0.843	0.877	0.891
	IoU	0.689	0.780	0.743	0.789	0.811
	Hausdorff (mm)	17.5	16.9	16.4	17.1	15.9

## Data Availability

The publicly shared prostate cancer dataset is available at https://www.kaggle.com/c/prostate-cancer-grade-assessment.
